# Messaging to Increase Public Support for Naloxone Distribution Policies in the United States: Results from a Randomized Survey Experiment

**DOI:** 10.1371/journal.pone.0130050

**Published:** 2015-07-01

**Authors:** Marcus A. Bachhuber, Emma E. McGinty, Alene Kennedy-Hendricks, Jeff Niederdeppe, Colleen L. Barry

**Affiliations:** 1 Center for Health Equity Research and Promotion, Philadelphia Veterans Affairs Medical Center, Philadelphia, PA, United States of America; 2 Leonard Davis Institute of Health Economics, University of Pennsylvania, Philadelphia, PA, United States of America; 3 Robert Wood Johnson Foundation Clinical Scholars Program, University of Pennsylvania, Philadelphia, PA, United States of America; 4 Department of Health Policy and Management, Johns Hopkins Bloomberg School of Public Health, Baltimore, MD, United States of America; 5 Department of Mental Health, Johns Hopkins Bloomberg School of Public Health, Baltimore, MD, United States of America; 6 Department of Communication, Cornell University, Ithaca, NY, United States of America; Penn State College of Medicine, UNITED STATES

## Abstract

**Background:**

Barriers to public support for naloxone distribution include lack of knowledge, concerns about potential unintended consequences, and lack of sympathy for people at risk of overdose.

**Methods:**

A randomized survey experiment was conducted with a nationally-representative web-based survey research panel (GfK KnowledgePanel). Participants were randomly assigned to read different messages alone or in combination: 1) factual information about naloxone; 2) pre-emptive refutation of potential concerns about naloxone distribution; and 3) a sympathetic narrative about a mother whose daughter died of an opioid overdose. Participants were then asked if they support or oppose policies related to naloxone distribution. For each policy item, logistic regression models were used to test the effect of each message exposure compared with the no-exposure control group.

**Results:**

The final sample consisted of 1,598 participants (completion rate: 72.6%). Factual information and the sympathetic narrative alone each led to higher support for training first responders to use naloxone, providing naloxone to friends and family members of people using opioids, and passing laws to protect people who administer naloxone. Participants receiving the combination of the sympathetic narrative and factual information, compared to factual information alone, were more likely to support all policies: providing naloxone to friends and family members (OR: 2.0 [95% CI: 1.4 to 2.9]), training first responders to use naloxone (OR: 2.0 [95% CI: 1.2 to 3.4]), passing laws to protect people if they administer naloxone (OR: 1.5 [95% CI: 1.04 to 2.2]), and passing laws to protect people if they call for medical help for an overdose (OR: 1.7 [95% CI: 1.2 to 2.5]).

**Conclusions:**

All messages increased public support, but combining factual information and the sympathetic narrative was most effective. Public support for naloxone distribution can be improved through education and sympathetic portrayals of the population who stands to benefit from these policies.

## Introduction

Between 1999 and 2013, the number of people dying from a drug overdose involving opioid analgesics in the United States more than quadrupled, from 4,030 to 16,235 [1]. More recently, an increase in heroin overdose deaths has been observed in several states, with some areas noting a marked transition from overdose deaths involving opioid analgesics to heroin overdoses [2–4]. Multiple strategies to address opioid overdose mortality have been proposed, including naloxone distribution programs [5].

Naloxone, an opioid antagonist, has long been used in medical settings and is highly effective in reversing the effects of an opioid overdose [6]. More recently, numerous programs across the United States have distributed naloxone, paired with education on how to identify, prevent, and treat overdoses, to the lay public (e.g., friends and family members of people who use opioids). Evaluations of these programs have consistently found that lay persons can accurately identify and respond to an opioid overdose with intranasal or intramuscular naloxone, preventing a fatal overdose [7–16]. According to a report by the Centers for Disease Control and Prevention, between 1996 and 2010 over 50,000 people in the United States were trained to use naloxone, resulting in over 10,000 reported overdose reversals [17]. On a community level, recent research has found strong associations between naloxone distribution programs and lower rates of opioid overdose fatalities [18]. In addition to naloxone distribution programs for friends and family members of people who use opioids, programs training first responders like police, firefighters, and emergency medical technicians who traditionally have not been trained to use naloxone, have also demonstrated positive results [19,20].

Despite evidence of safety and efficacy, naloxone distribution programs, like other harm reduction interventions, face logistical and ideological challenges [21,22]. First, individuals who witness an overdose may not call for medical attention or administer naloxone because of legal concerns [23–25]. Medical providers that prescribe naloxone may have similar concerns [25–27]. To address this issue, laws protecting individuals who call for medical attention for an overdose as well as laws protecting individuals who administer, and providers who prescribe, naloxone have been enacted in several states [26,28,29]. Second, some critics argue that giving naloxone to people who use opioids could potentially lead to increased use because people will rely on naloxone to rescue them from a life-threatening overdose [21]; however, this concern is not supported by previous research. In fact, some research suggests that opioid use may be reduced after participating in a naloxone distribution program as these programs provide education on how to prevent an overdose [8,10,12]. Third, some critics argue that preventing overdose mortality is futile because people who use opioids will continue using and overdose again. While opioid use disorder is a chronic medical illness with periods of remission and relapse, treatment does improve outcomes [30–33], and individuals who experience a fatal overdose have lost the opportunity to engage in it.

Although data on public opinion about naloxone distribution programs are lacking, there are many potential reasons why naloxone distribution may have a low rate of public support. Stigma and negative public opinion around drug use and people who use drugs are barriers to support of a wide range of public policies such as insurance parity, housing support, and job support [34]. More specific to naloxone distribution, lack of familiarity with naloxone, concerns about the unintended consequences of naloxone, and lack of compassion or sympathy for people who use drugs may lead to low public support. Previous research shows that public opinion is a substantial contributor to the enactment of public policy [35]. For example, stigma and negative public opinion toward people who use drugs are connected to the criminalization of drug use and enactment of punitive criminal justice-focused policies [36]. Improving public support for naloxone distribution may contribute to wider implementation.

If there is a general gap in knowledge about the effects of naloxone distribution, factual information about the safety and efficacy of naloxone and pre-emptive refutation of potential objections to naloxone distribution might improve public support [37]. In addition, as many Americans view drug use as a moral failing [36], overdose may be viewed as the predictable consequence of poor choices as opposed to the result of a medical condition warranting public health intervention; presenting narratives which evoke emotional responses such as sadness or sympathy may improve public support for naloxone distribution in this case [38,39]. To examine the effects of different types of messages to improve public support for, and foster positive beliefs about, naloxone distribution in addition to other policies aimed at reducing opioid overdose mortality, we conducted a randomized survey experiment. We hypothesized that provision of factual information alone would increase support for naloxone distribution, but it would also increase negative beliefs about the unintended consequences of naloxone distribution. We also hypothesized that pre-emptive refutation of these concerns would prevent increases in negative beliefs. Finally, we hypothesized that a sympathetic narrative about naloxone’s benefits would also bolster support, and the pairing of factual information with a sympathetic narrative would be most effective at increasing support for naloxone distribution policies.

## Methods

A randomized survey experiment was fielded September 18^th^ through October 13^th^, 2014, using the nationally-representative web-based GfK survey research panel (KnowledgePanel). The survey panel consists of about 55,000 adult members ages 18 and older who were recruited through address-based sampling using a frame of residential addresses that covers approximately 97% of US households [40]. Recruited households without Internet access were provided with laptop computers. Newly recruited panel members complete a demographic profile including information such as gender, age, race/ethnicity, income, education. Survey participation was rewarded with a variety of small incentives (small cash awards, gift prizes, sweepstakes opportunities), and on average, panel members participate in about two surveys per month. In the current study, invitations to participate did not include information about survey content. To account for nonresponse and error from panel recruitment methods or panel attrition, survey weights were calculated and provided by GfK along with survey responses. The GfK panel has been used extensively for survey research in diverse academic disciplines [41–45].

### Message Content

We tested the effects of three different types of persuasive messages on public support for naloxone distribution programs, alone or in combination: (1) factual information (text about opioid analgesic overdoses and evidence on the safety and efficacy of naloxone in preventing overdose death); (2) pre-emptive refutation (text providing counterarguments to two common concerns around naloxone distribution, that having access to naloxone will lead to more overdoses because people will believe they can be “rescued” and that people who overdose and are saved with naloxone will just continue using and overdose again); and a (3) sympathetic narrative (text about a mother who struggles with her daughter’s addiction to opioid analgesics and subsequent fatal overdose). The factual information message was 13 sentences in length, the pre-emptive refutation message was 8 sentences in length, and the sympathetic narrative was 20 sentences in length. The sympathetic narrative was longer than the factual information and pre-emptive refutation messages because effective stories require contextual information to humanize characters and offer a compelling storyline. Prior to reading the randomized message(s), all participants read a brief definition of opioid analgesics—termed “prescription pain medication” throughout the survey to be more accessible to participants—which included a link to view a medication list (S1 Appendix). The factual information read as follows:

In 2011, nearly 17,000 people died from prescription pain medication overdoses in the United States—equal to 46 deaths per day. Over the last decade, the number of prescription pain medication overdose deaths has increased by more than 300 percent. Government officials, medical experts, and community leaders have declared prescription pain medication overdoses a national crisis.

Naloxone is a medicine that is very effective at saving lives by reversing life threatening overdoses of prescription pain medication. Naloxone can be given by injection or nasal spray. Medical experts believe naloxone is so safe that anyone can be trained to administer it, even friends and family members of people at risk of overdose and first responders like police officers and firefighters. If naloxone is mistakenly given to someone who is not having a prescription pain medication overdose, there are no bad side effects.

Most overdoses occur among people who are at home with friends or family. If these friends or family members had naloxone, they could administer it to potentially save the life of a person overdosing. When someone overdosing is using prescription pain medication illegally, friends and family are often afraid to call the police because they don’t want to be arrested and put in jail for being around someone using drugs illegally. Even if they do call, most police officers and firefighters do not have naloxone and have to wait for an ambulance to arrive. A person overdosing may die before getting the naloxone treatment he or she needs. Providing training and naloxone medication to friends and family members of people at risk of overdose and first responders like police officers and firefighters could save thousands of lives every year.

The pre-emptive refutation message read as follows:

Some people don't believe that the lives of people overdosing on prescription pain medication are worth saving. They say that using naloxone to save a person overdosing is pointless because the person will just continue using prescription pain medication and eventually overdose again. Some people also say that giving naloxone to people who are addicted to prescription pain medication will just cause them to use more, because they will think of naloxone as a “safety net” to save them from an overdose.

But in fact, many people who overdose and are saved because of naloxone will see it as a wake-up call and enter treatment for their addiction. Letting someone die from an overdose that could be prevented is cruel and misguided, especially because naloxone is such a safe medication. Friends and family members of people addicted to prescription pain medication often feel helpless watching their loved one struggle, but providing naloxone to these friends and family members is giving them the power to save a life. And providing naloxone to first responders like police officers and firefighters allows them to be better prepared to help when they arrive at the scene of an overdose. People saved from a prescription pain medication overdose can recover and go on to live long, productive, and healthy lives.

The sympathetic narrative read as follows:

Mother’s Day has become a very difficult time for Mary since she lost her daughter, Erika, to an overdose of prescription pain medication two years ago.

It all started after Erika was hit by a car while driving home from college. Left with back, hip, and knee injuries and severe pain, Erika turned to doctors for help. She started physical therapy and her doctor prescribed Percocet, Vicodin, and OxyContin—strong prescription pain medications—to help ease her pain.

For the first few months, things were going well. Erika was recovering her ability to get around and was catching up on her school work. But then, Mary saw something change. Erika started taking more prescription pain medication. When her prescription ran out and the doctor would not give her another, she started getting old prescriptions from friends. Mary suspected that Erika had developed an addiction to prescription pain medication and tried to convince her to get help. At first Erika said she wasn’t addicted and didn’t need help, but after a few more months she admitted to her mother that she had a problem. Even though Erika was willing to get help, Mary couldn’t find an addiction treatment program that was seeing new patients anywhere in her community. She finally found a clinic nearly two hours away that could treat Erika, but the first available appointment was several weeks away.

Mary scheduled the appointment, but a few days later she came home from work and found Erika on the bathroom floor, barely breathing. Mary called 911, but by the time Erika got to the hospital it was too late and she was pronounced dead from an overdose of pain medication.

Thinking back, Mary wishes she had known about naloxone, a medication she could have been trained to use in an emergency that helps people who are overdosing. Naloxone could have saved her daughter’s life. Mary recently got trained to use naloxone and started a support and education group for parents who have children that are addicted to prescription pain medication. She has also been pushing for the local police and fire departments in her town to train first responders to carry naloxone medication in case they arrive at the scene of an overdose before paramedics. On top of her full-time job, Mary has been working tirelessly to prevent overdose deaths in her community because she believes no parent should have to go through losing a child the way she did.

We randomized survey participants to a no-exposure control group or to read one of five message combinations: (1) factual information only, (2) factual information plus pre-emptive refutation, (3) sympathetic narrative only, (4) sympathetic narrative plus factual information, or (5) all three messages in combination.

### Outcome Measures

We examined effects of randomized condition on two main categories of outcomes created by the study team: support for naloxone distribution and other overdose mortality prevention policies and beliefs about naloxone. For naloxone distribution and overdose mortality prevention policies, we asked participants whether they support or oppose:1) training first responders like police officers and firefighters to use naloxone in cases when they arrive at the scene before paramedics; 2) providing naloxone to friends and family members of people using opioid analgesics; 3) passing laws to protect people who call for medical help when they themselves or someone else is experiencing an overdose; 4) passing laws to provide legal protection for people giving naloxone to a friend or family member; and 5) increasing government spending to improve screening and treatment of opioid addiction. For beliefs about naloxone, we asked if participants agreed or disagreed that: 1) providing naloxone to first responders, like police officers and firefighters, would save lives; 2) providing naloxone to friends and family members of people who use opioid analgesics would save lives; 3) distributing naloxone will encourage people to use even more opioid analgesics because they will assume they can be saved from a life-threatening overdose; 4) preventing overdoses is ineffective because people with opioid addiction will continue to use and eventually overdose again; and 5) naloxone is a medication that should only be given by medical professionals.

We randomized the order of policy and belief question blocks for each survey participant, as well as the order of questions within the blocks, to cancel out any effects of question order on responses. We asked each question using seven-point scales anchored at 1 (strongly oppose), 4 (neither oppose nor favor), and 7 (strongly favor) for policy questions and 1 (strongly disagree), 4 (neither agree nor disagree), and 7 (strongly agree) for belief questions. We did not include a “don’t know” option, but participants could choose not to answer a given question. For policy questions, we dichotomized responses into support (5–7) or do not support (1–4) and for belief questions, we dichotomized responses into agree (5–7) or do not agree (1–4). Responses were dichotomized in this way as 50% is a meaningful cut-off for policy support (majority support) and thus modeling the outcomes dichotomously allowed for estimation of the predicted probabilities of majority support. We excluded survey responses if participants completed the survey in fewer than 1.5 minutes (1 minute for the no message control group) or greater than 240 minutes. Of the 2,321 panel members invited to respond, 1,685 completed the survey for a completion rate of 72.6%. Among participants, 5.2% (87/1,685) were excluded for not completing the survey in the specified time window.

### Statistical Analysis

To assess the representativeness of survey participants, we examined socio-demographic characteristics (age, gender, race/ethnicity, education level, employment status, geographic region of residence) using the un-weighted and weighted responses in comparison to national estimates from the March 2013 Current Population Survey. Characteristics of the study sample in terms of gender, age, race, education level, household income, employment status and region were close to the national comparison (Table 1). There was an overrepresentation in the unweighted study sample of white people (+ 7.5%), unemployed people (+ 2.6%), and those age 55 to 64 years (+ 5.9%). Next, to ensure randomization was successful at producing balanced groups, we compared the socio-demographic characteristics of participants in each survey arm using chi-square tests. Randomization to the experimental conditions resulted in six well balanced groups without any significant differences in the above mentioned characteristics (Table 2). Finally, we calculated the percentage of participants supporting each policy item or agreeing with each belief, along with 95% confidence intervals (95% CI). The item response rate was 99.5% and for a given question, participants with missing responses were excluded from that question but included in all other questions for which data were available.

**Table 1:** Un-weighted and weighted characteristics of survey participants compared with national rates (N = 1,598).

	Un-weighted	Weighted	National Comparison
Women (%)	50.1	52.0	51.9
Age (%)			
Ages 18–24	10.1	12.3	12.7
Ages 25–34	16.2	17.6	17.5
Ages 35–44	15.8	16.3	16.8
Ages 45–54	16.5	16.1	18.4
Ages 55–64	22.2	20.4	16.3
Age 65 +	19.2	17.3	18.3
Race (%)			
White only	73.5	66.1	66.0
Black only	9.4	11.5	11.6
Other	17.2	22.5	22.5
Hispanic ethnicity (%)	10.0	15.1	15.0
Education (%)			
< High school degree	9.9	12.4	12.6
High school degree	31.5	29.8	29.6
Some college	27.6	28.9	28.9
Bachelor's degree or higher	31.0	28.9	28.9
Household income (%)			
Under $10,000	5.3	5.8	5.2
$10,000–24,999	12.9	11.9	13.3
$25,000–49,999	21.8	22.7	22.7
$50,000–74,999	19.2	18.1	18.4
$75,000 or higher	40.9	41.6	40.5
Employment status (%)			
Employed	57.2	57.9	59.9
Unemployed	7.5	8.2	4.9
Retired	19.1	16.9	17.2
Other (e.g., disabled, homemaker, other)	16.3	17.0	18.1
Region (%)			
Northeast	18.5	17.8	18.2
Midwest	23.2	21.7	21.4
South	36.1	37.1	37.1
West	22.2	23.5	23.4

Note: GfK sample weights used to calculate descriptive statistics. For socio-demographic characteristics, comparison data extracted from the March 2013 Current Population Survey.

**Table 2:** Comparison of the characteristics of survey participants randomized to each message exposure (N = 1,598).

	No-exposure control (n = 267)	Factual information (n = 260)	Factual information plus refutation (n = 266)	Sympathetic narrative (n = 264)	Sympathetic narrative plus factual information (n = 276)	Sympathetic narrative plus factual information plus refutation (n = 265)	P
Women (%)	52.6	51.3	51.8	51.1	52.2	52.9	0.99
Age (%)							0.98
Ages 18–24	13.1	13.5	12.2	10.5	11.5	13.3	
Ages 25–34	15.9	15.3	17.1	21.4	19.0	16.9	
Ages 35–44	17.9	18.1	16.4	14.5	16.5	14.6	
Ages 45–54	15.8	15.3	15.7	14.5	18.1	16.9	
Ages 55–64	19.1	21.1	21.3	24.1	18.4	18.7	
Age 65 +	18.2	16.8	17.3	15.0	16.6	19.7	
Race (%)							1.0
White only	64.8	65.5	66.2	66.9	66.9	66.1	
Black only	11.8	11.3	11.2	11.7	11.5	11.2	
Other	23.5	23.2	22.6	21.4	21.7	22.7	
Hispanic ethnicity (%)	15.5	15.4	14.8	14.8	15.2	14.9	0.99
Education (%)							1.0
< High school degree	12.7	12.2	12.0	12.9	12.2	12.4	
High school degree	29.5	29.6	30.4	29.4	29.9	30.1	
Some college	29.3	29.0	28.1	27.5	29.4	30.0	
Bachelor's degree or higher	28.6	29.2	29.5	30.3	28.5	27.5	
Household income (%)							0.99
Under $10,000	6.1	4.8	3.9	6.9	6.1	7.2	
$10,000–24,999	11.8	13.3	14.0	10.4	11.1	10.8	
$25,000–49,999	22.2	22.0	22.6	22.9	22.9	23.3	
$50,000–74,999	18.1	18.6	18.0	18.6	18.7	16.6	
$75,000 or higher	41.8	41.3	41.6	41.2	41.3	42.1	
Employment status (%)							0.48
Employed	56.3	59.6	62.3	55.5	59.2	54.3	
Unemployed	6.2	7.2	9.5	9.4	6.3	10.9	
Retired	16.8	17.0	16.4	15.2	17.3	18.8	
Other (e.g., disabled, homemaker, other)	20.8	16.2	11.9	19.9	17.2	16.0	
Region (%)							1.0
Northeast	17.6	18.1	17.8	18.9	16.8	17.3	
Midwest	21.8	21.3	21.6	22.2	22.2	21.2	
South	37.9	37.1	36.7	36.3	37.5	36.8	
West	22.7	23.6	23.8	22.7	23.5	24.7	

To test the effect of each message exposure compared with the no-exposure control group, we then created logistic regression models for each outcome question. In each model, the dependent variable was support for a specific policy or agreement with a specific belief and the main independent variable was the randomized message exposure. Using the dichotomized measures of policy support and agreement with beliefs, we constructed odds ratios (OR) with 95% CIs to compare the effect of each message exposure to the no-exposure control group. Next, to examine the effects of adding the sympathetic narrative to factual information, we used similar logistic regression models to compare the effect of exposure to the sympathetic narrative plus factual information, using participants exposed to factual information alone as the referent group. As survey participants were randomly assigned to message exposures, we did not include covariates in regression models [46]. We conducted all statistical analyses using Stata 13.1(StataCorp LP, College Station, TX, USA).

### Sensitivity analysis

As a sensitivity analysis, we created separate policy support and belief scales from the policy questions and the belief questions, respectively. We used Crohnbach’s alpha to assess consistency of the items and used factor analysis to identify candidates for deletion. After reverse coding the negatively worded questions, we averaged responses to policy questions and belief questions to create the scales. Finally, we created linear regression models to determine the effect of different messages on the scales. For each model, the policy support or belief scale was the dependent variable and the message exposure was the main independent variable.

### Ethics Statement

This study was determined to be exempt by the Johns Hopkins Bloomberg School of Public Health Institutional Review Board.

## Results

### Support for Naloxone Distribution and Other Policies in the No-Exposure Control Group

The first column of Table 3 indicates support for overdose mortality prevention policies among the no-exposure control group. Public support was strongest for training first responders to use naloxone (63.2%) and passing laws to protect people if they call for medical help for an overdose (52.4%). Fewer participants in the no-exposure control group supported policies to protect people from legal action if they administer naloxone to friends or family members (41.6%) or increasing government spending to improve addiction screening and treatment (38.5%). Public support was lowest for the policy to provide naloxone to friends and family members of people who use opioid analgesics (24.4%).

**Table 3:** Effects of messages on support for naloxone distribution and other overdose mortality prevention policies compared to the no-exposure control group.

	Message Exposure					
Policy^a^	No-exposure control (n = 267)	Factual information (n = 260)	Factual information plus refutation (n = 266)	Sympathetic narrative (n = 264)	Sympathetic narrative plus factual information (n = 276)	Sympathetic narrative plus factual information plus refutation (n = 265)
Train first responders to use naloxone (n = 1592)	63.2% (56.9 to 69.1)	77.4% (71.2 to 82.6)**	79.0% (73.3 to 83.7)***	80.5% (74.7 to 85.2)***	87.4% (82.5 to 91.1)***	85.7% (80.0 to 89.9)***
Provide naloxone to friends and family members (n = 1592)	24.4% (19.4 to 30.4)	45.5% (39.2 to 55.4)***	49.0% (42.7 to 55.4)***	40.1% (34.2 to 46.4)***	62.6% (56.4 to 68.4)***	64.3% (57.6 to 70.5)***
Pass laws to protect people if they call for medical help for an overdose (n = 1592)	52.4% (45.9 to 58.7)	53.3% (46.8 to 59.8)	59.0% (52.6 to 62.2)	58.0% (51.7 to 64.1)	66.5% (60.2 to 72.2)**	69.2% (62.7 to 75.0)***
Pass laws to protect people if they give naloxone (n = 1592)	41.6% (35.4 to 48.0)	58.0% (51.4 to 64.3)***	58.6% (52.1 to 64.8)***	55.6% (49.3 to 61.7)**	67.7% (61.5 to 73.3)***	69.1% (62.4 to 75.0%)***
Increase government spending to improve addiction screening and treatment (n = 1587)	38.5% (32.3 to 45.0)	33.9% (28.1 to 40.2)	34.3% (28.5 to 40.6)	47.9% (41.7 to 54.2)*	50.6% (44.3 to 56.9)**	43.2% (36.7 to 49.8)

*P ≤ 0.05 compared to the no-exposure control group using logistic regression

** P ≤ 0.01 compared to the no-exposure control group using logistic regression

*** P ≤ 0.001 compared to the no-exposure control group using logistic regression

^a^Percent support for policies calculated as percentage of sample responding 5, 6, or 7 on the seven point scale for each measure (i.e., somewhat favor, favor, or strongly favor)

### Effects of Messages on Support for Naloxone Distribution and Other Policies

**Fig 1:**
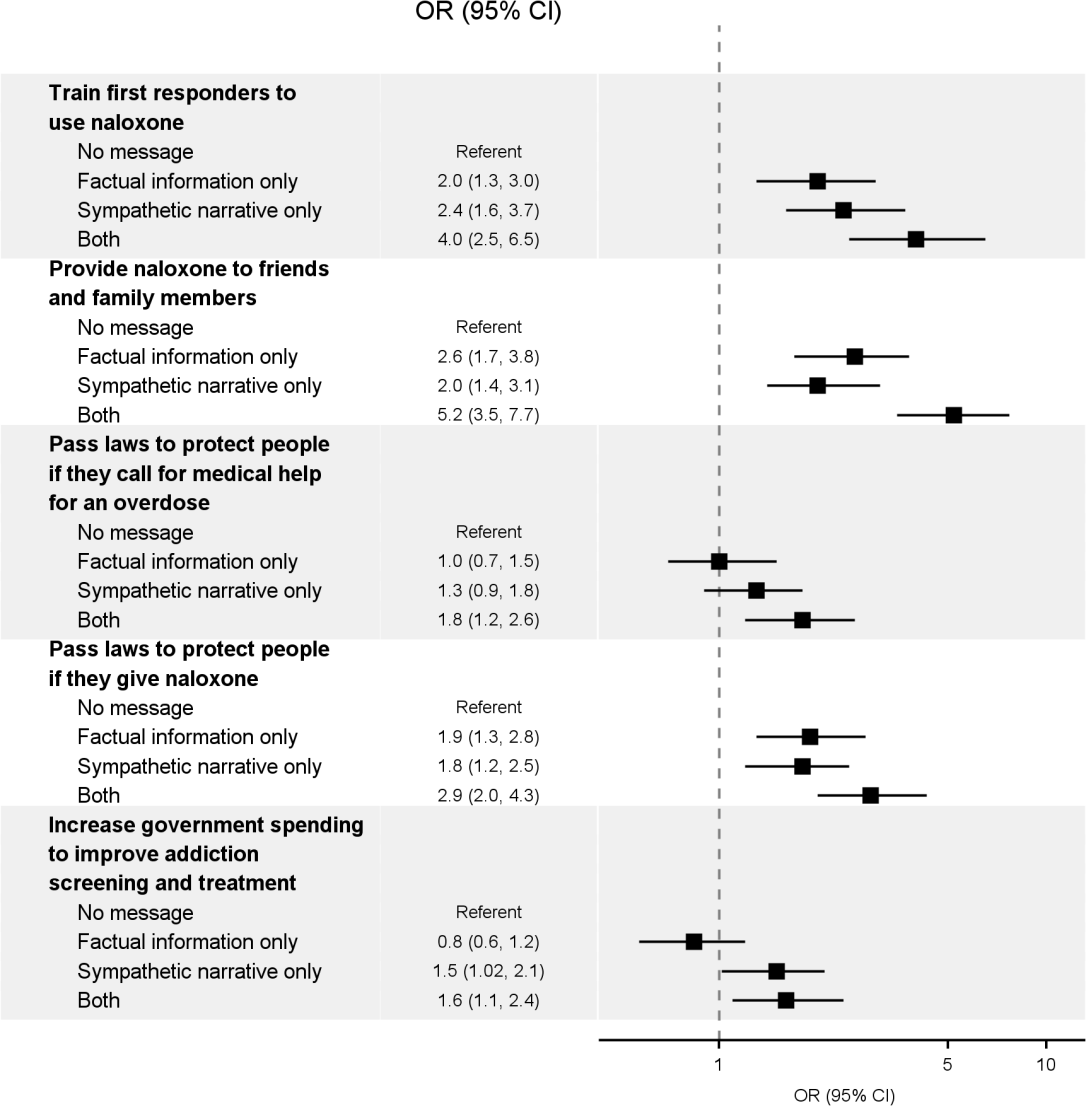
Effects of factual information, a sympathetic narrative, or both on support for naloxone distribution and other opioid overdose mortality prevention policies.

Several message exposures led to significantly higher support for policies compared with the no-exposure control group (Table 3, Fig 1 and S1 Fig). Factual information alone led to higher support for training first responders to use naloxone (77.4% versus 63.2% in the no-exposure control group), providing naloxone to friends and family members of people using opioids (45.5% versus 24.4% in the no-exposure control group), and passing laws to protect people who administer naloxone (58.0% versus 41.6% in the no-exposure control group). The sympathetic narrative alone also led to higher support for training first responders to use naloxone (80.5% versus 63.2% in the no-exposure control group), providing naloxone to friends and family members of people using opioids (40.1% versus 24.4% in the no-exposure control group), passing laws to protect people who administer naloxone (55.6% versus 41.6% in the no-exposure control group), and increasing government spending to improve addiction screening and treatment (47.9% versus 38.5% in the no-exposure control group). Results from the sensitivity analysis using a policy support scale were similar (S2 Appendix).

Directly comparing the effects of adding the sympathetic narrative to factual information, using participants exposed to factual information alone as the referent group, we found that the combination led to higher support for all policies: providing naloxone to friends and family members (OR: 2.0 [95% CI: 1.4 to 2.9]), training first responders to use naloxone (OR: 2.0 [95% CI: 1.2 to 3.4]), passing laws to protect people if they administer naloxone (OR: 1.5 [95% CI: 1.04 to 2.2]), passing laws to protect people if they call for medical help for an overdose (OR: 1.7 [95% CI: 1.2 to 2.5]), and increasing government spending to improve addiction screening and treatment (OR: 2.0 [95% CI: 1.4 to 2.9]).

### Beliefs about Naloxone in the No-Exposure Control Group

The first column of Table 4 shows agreement with beliefs about naloxone among participants in the no-exposure control group. A majority believed that providing naloxone to first responders would save lives (60%); however, only 35.9% believed that providing naloxone to friends and family members would save lives. A similar percentage held negative beliefs about naloxone: 31.4% thought that distributing it will encourage people to use more opioid analgesics and 39.0% believed that preventing overdoses is ineffective because people will just continue to use and overdose again. Almost half of participants believed that naloxone should only be given by medical professionals (45.8%).

**Table 4:** Effects of messages on beliefs about naloxone distribution compared to the no-exposure control group.

	Message Exposure					
Belief^a^	No-exposure control (n = 267)	Factual information (n = 260)	Factual information plus refutation (n = 266)	Sympathetic narrative (n = 264)	Sympathetic narrative plus factual information (n = 276)	Sympathetic narrative plus factual information plus refutation (n = 265)
Providing naloxone to first responders would save lives. (n = 1587)	60.0% (53.5 to 66.1)	73.7% (67.3 to 79.1)**	76.1% (70.0 to 81.3)***	77.0% (71.2 to 82.0)***	88.4% (83.5 to 91.9)***	83.5% (77.6 to 88.1)***
Providing naloxone to friends and family members would save lives (n = 1590)	35.9% (30.0 to 42.3)	55.8% (49.2 to 62.3)***	58.6% (52.5 to 65.0)***	58.8% (52.5 to 64.9)***	73.9% (67.9 to 79.1)***	72.9% (66.5 to 78.6)***
Distributing naloxone will encourage people to use even more opioid analgesics (n = 1591)	31.4% (25.6 to 37.7)	41.9% (35.6 to 48.5)*	28.1% (22.7 to 34.1)	31.6% (26.0 to 37.8)	34.8% (29.0 to 41.1)	26.9% (21.2 to 33.5)
Preventing overdoses is ineffective because people will overdose again (n = 1589)	39.0% (33.0 to 45.4)	48.0% (41.5 to 54.5)	30.2% (24.7 to 36.4)*	31.0% (25.4 to 37.1)	32.0% (26.4 to 38.2)	22.2% (16.9 to 28.6)***
Naloxone should only be given by medical professionals (n = 1591)	45.8% (39.5 to 52.2)	34.5% (28.5 to 41.0)*	35.2% (29.3 to 41.6)*	40.2% (34.1 to 46.6)	25.8% (20.6 to 31.7) ***	24.3% (18.6 to 30.9)***

*P ≤ 0.05 compared to the no-exposure control group using logistic regression

** P ≤ 0.01 compared to the no-exposure control group using logistic regression

*** P ≤ 0.001 compared to the no-exposure control group using logistic regression

^a^Percent agreeing with beliefs calculated as percentage of sample responding 5, 6, or 7 on the seven point scale for each measure (i.e., somewhat agree, agree, or strongly agree)

### Effects of Messages on Naloxone Beliefs

**Fig 2:**
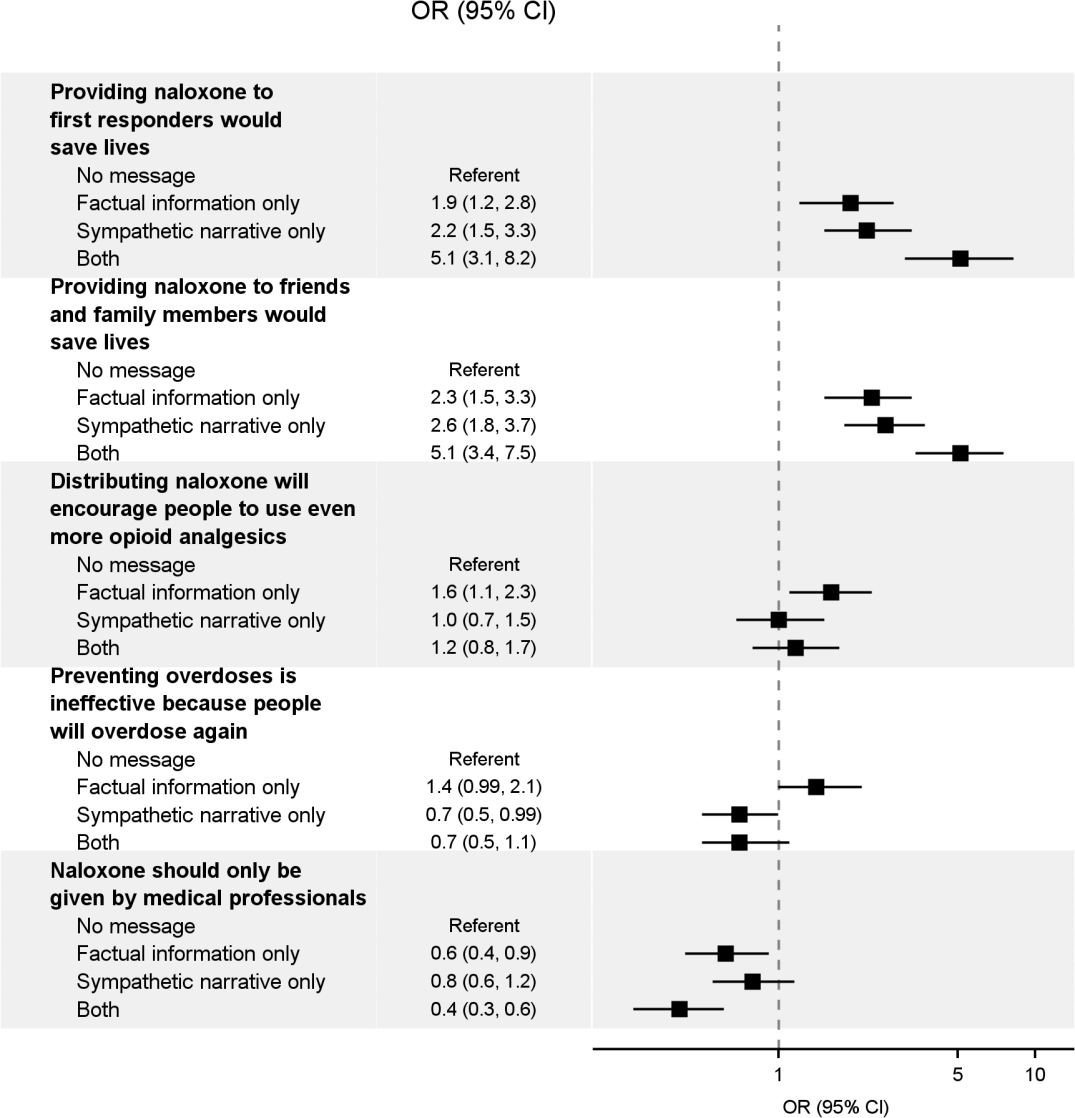
Effects of factual information, a sympathetic narrative, or both on beliefs about naloxone distribution.

In almost all cases, participants exposed to messages about naloxone had significantly different reported beliefs than those in the no-exposure control group (Table 4, Fig 2 and S2 Fig). Factual information alone led to more participants believing that providing naloxone to first responders would save lives (73.7% versus 60.0% in the no-exposure control group) and providing naloxone to friends and family members of people who use opioids would save lives (55.8% versus 35.9% in the no-exposure control group). Similarly, the sympathetic narrative alone led to more participants believing that providing naloxone to first responders (77.0% versus 60.0% in the no-exposure control group) and providing naloxone to friends and family members of people who use opioids (58.8% versus 35.9% in the no-exposure control group) would save lives. Participants in all message groups including factual information (i.e., alone or in combination with refutation and the sympathetic narrative) were less likely to believe that naloxone should only be given by medical professionals compared with participants in the no-exposure control group. Results from the sensitivity analysis using a belief scale were similar (S2 Appendix).

Provision of factual information alone led to higher agreement that distributing naloxone will encourage people to use more opioid analgesics compared with the no-exposure control group (41.9% versus 31.4%, respectively); this was not seen in the factual information plus refutation group (28.1% versus 31.4%). In addition, participants receiving factual information, compared with the no-exposure control group, were similarly likely to believe that preventing overdoses is ineffective because people will overdose again (48.0% versus 39.0%, respectively), but those receiving factual information plus refutation were less likely to believe this (30.2% versus 39.0%).

Directly comparing the effects of adding the sympathetic narrative to factual information, using participants exposed to factual information alone as the referent group, we found that the combination led to a higher percentage of participants who believed that providing naloxone to first responders (OR: 2.7 [95% CI: 1.6 to 4.5]), and to friends and family members of people using opioid analgesics (OR: 2.2 (95% CI: 1.5 to 3.3]), would save lives. These participants were also less likely to agree that preventing overdoses is ineffective (OR: 0.5 [95% CI: 0.4 to 0.7]) and that naloxone should only be given by medical professionals (OR: 0.6 [95% CI: 0.4 to 0.99]).

## Discussion

In a randomized, nationally-representative survey experiment, we found that public support for naloxone distribution policies was substantially higher after exposure to several different types of messages designed to educate participants about naloxone, refute common arguments against naloxone distribution, and evoke a sympathetic reaction toward people who use opioids. Our findings suggest that while most Americans do not currently support all naloxone distribution policies, there is an opportunity to improve public support through education and sympathetic portrayals of the population who stands to benefit from these policies.

Compared to the control group, exposure to factual information alone led to higher support for providing naloxone to first responders as well as friends and family members of people who use opioids, suggesting that the general public has little knowledge of the safety and efficacy of naloxone. While the medication has been in routine clinical use for many years, it was not in widespread use in a “take-home” form to treat opioid overdoses until relatively recently. Furthermore, the concept of using a prescription medication to block an overdose of another prescription medication may be confusing; people may wonder if naloxone itself is a controlled substance with potential for recreational use, or think of it as analogous to methadone, an opioid agonist used to treat opioid use disorder, which also has the potential for overdose. Our finding that factual information alone can significantly increase support for naloxone distribution policies suggests that educating the public about naloxone’s safety and efficacy will play an important role in garnering public support.

While factual information alone increased support for several policies relative to no exposure, it also increased negative beliefs about naloxone. Participants receiving factual information only were significantly more likely to agree that distributing naloxone will encourage people to use even more opioid analgesics, and more likely to believe that preventing overdoses is ineffective, although this second comparison did not reach statistical significance. Learning more about how naloxone works potentially prompted some participants to think of ways it could be misused or be more receptive when those unintended consequences were presented to them. However, by adding a pre-emptive refutation message to factual information, those negative beliefs were mitigated. Future efforts to educate the public about the safety and efficacy of naloxone should pre-emptively address potential concerns about the unintended effects of naloxone distribution.

The combination of a sympathetic narrative plus factual information appeared to be the most effective messaging approach for promoting support for naloxone distribution policies. Not only did this combination lead to significantly higher support for all policies relative to no message exposure, but it also led to higher support than that seen in participants receiving factual information alone. The percentage agreeing that providing naloxone to friends and family members would save lives approximately doubled, and there was no increase in negative beliefs about naloxone. Therefore, where possible, efforts to improve public support for overdose mortality prevention policies through education should also incorporate personal narratives to increase persuasiveness.

Our finding that the sympathetic narrative was persuasive, both in isolation and over and above the provision of factual information about naloxone, has several potential explanations. Previous work has shown that messages evoking certain emotions (e.g., sympathy or sadness) for an individual representing a broader population can lead people to support policies that benefit that population [38]. This may be particularly important in the context of naloxone distribution to people who use opioids. As drug addiction is viewed as a moral failing by the majority of Americans, people who use drugs are often perceived very negatively [34,36]. Furthermore, harm reduction interventions (e.g., syringe exchange programs) have a long history of being highly controversial with the American public [47–49]. Messages evoking sympathetic reactions may overcome these negative attitudes toward people who use drugs and harm reduction interventions for them. In particular, results from the current study suggest that sympathetic portrayals of individuals whose lives are affected by others’ drug addiction (e.g., the mother in our sympathetic narrative) may be successful in shaping support for harm reduction interventions by focusing on the dramatic consequences of overdose to non-users.

### Limitations

This study has several limitations. First, the exposure to the messages tested in in this Internet-based experiment may differ from the typical media environment where individuals are exposed to or select information about opioid analgesic overdoses and overdose prevention policies; our findings may not be representative of the impacts of real media content produced to convey these messages, however, themes of our messages were drawn from a convenience sample of actual news stories. Second, we tested only immediate responses to the messages, so the durability and stability of the assessed opinions is unclear. In addition, prior knowledge or experience with naloxone was not assessed and participants in the no-exposure control group, who were given no information about naloxone, may have drawn upon limited or no prior knowledge about the topic when answering survey questions. Third, we may not have identified or included other important messages which would change policy support and beliefs about naloxone in significant ways, such as news stories or video messages. Fourth, from the current analysis, we cannot exclude that differential effects of messages may be due to differences in content (e.g., information versus sympathetic narrative) as well as differences in other factors (e.g., word length). Finally, web-based studies are vulnerable to sampling biases. The GfK panel attempts to minimize such concerns by using address-based sampling to include households without a landline telephone. Furthermore, invitations to participate in surveys do not contain information about the content, so it is unlikely that participants chose whether or not to participate based on their interest in the topic.

## Conclusions

In summary, support for overdose mortality prevention policies was substantially higher among participants exposed to several different types of messages. Our findings that factual information alone can greatly improve support suggest that educating the public will be key in future efforts to widen naloxone distribution. Furthermore, education should be paired with pre-emptive refutation of concerns about potential unintended consequences of naloxone distribution. Finally, we found that a sympathetic narrative also improved public support, suggesting that sympathetic portrayals of people who use opioids through focusing on the loss faced by their family members is also effective in engendering support for naloxone distribution. Further research to determine which types of messages are optimal for subgroups of the population (e.g., by political ideology or education) will be useful in tailoring messages to increase persuasiveness. While a minority of Americans currently supports some of the overdose mortality prevention policies we studied, the results of our study provide public health officials and advocates with several effective tools to garner public support.

## Supporting Information

S1 Appendix Introductory text for the randomized survey experiment providing examples of opioid analgesics. (DOCX)

S2 Appendix Development of a policy support and belief scale and effects of different message exposures on scaled outcomes. (DOCX)

S1 Fig Effects of messages on support for naloxone distribution and other overdose mortality prevention policies. (TIF)

S2 Fig Effects of messages on beliefs about naloxone distribution. (TIF)

## References

[1] Hedegaard H, Chen LH, Warner M. Drug-poisoning deaths involving heroin: United States, 2000–2013. NCHS Data Brief 2015 3;(190):1–7. 25932890

[2] Dasgupta N, Creppage K, Austin A, Ringwalt C, Sanford C, Proescholdbell SK. Observed transition from opioid analgesic deaths toward heroin. Drug Alcohol Depend 2014 12 1;145:238–41. 10.1016/j.drugalcdep.2014.10.005 25456574

[3] Rudd RA, Paulozzi LJ, Bauer MJ, Burleson RW, Carlson RE, Dao D, et al Increases in heroin overdose deaths—28 States, 2010 to 2012. MMWR Morb Mortal Wkly Rep 2014 10 3;63(39):849–54. 25275328 PMC4584873

[4] Cicero TJ, Ellis MS, Surratt HL, Kurtz SP. The changing face of heroin use in the United States: a retrospective analysis of the past 50 years. JAMA Psychiatry 2014 7 1;71(7):821–6. 10.1001/jamapsychiatry.2014.366 24871348

[5] Trust for America's Health. Prescription Drug Abuse: Strategies to Stop the Epidemic 2013. Washington, DC; 2013 Oct.

[6] Strang J, Darke S, Hall W, Farrell M, Ali R. Heroin overdose: the case for take-home naloxone. BMJ 1996 6 8;312(7044):1435–6. 8664611 10.1136/bmj.312.7044.1435 PMC2351168

[7] Doe-Simkins M, Walley AY, Epstein A, Moyer P. Saved by the nose: bystander-administered intranasal naloxone hydrochloride for opioid overdose. Am J Public Health 2009 5;99(5):788–91. 10.2105/AJPH.2008.146647 19363214 PMC2667836

[8] Maxwell S, Bigg D, Stanczykiewicz K, Carlberg-Racich S. Prescribing naloxone to actively injecting heroin users: a program to reduce heroin overdose deaths. J Addict Dis 2006;25(3):89–96. 16956873 10.1300/J069v25n03_11

[9] Piper TM, Stancliff S, Rudenstine S, Sherman S, Nandi V, Clear A, et al Evaluation of a naloxone distribution and administration program in New York City. Subst Use Misuse 2008;43(7):858–70. 10.1080/10826080701801261 18570021

[10] Seal KH, Thawley R, Gee L, Bamberger J, Kral AH, Ciccarone D, et al Naloxone distribution and cardiopulmonary resuscitation training for injection drug users to prevent heroin overdose death: a pilot intervention study. J Urban Health 2005 6;82(2):303–11. 15872192 10.1093/jurban/jti053 PMC2570543

[11] Tobin KE, Sherman SG, Beilenson P, Welsh C, Latkin CA. Evaluation of the Staying Alive programme: training injection drug users to properly administer naloxone and save lives. Int J Drug Policy 2009 3;20(2):131–6. 10.1016/j.drugpo.2008.03.002 18434126

[12] Wagner KD, Valente TW, Casanova M, Partovi SM, Mendenhall BM, Hundley JH, et al Evaluation of an overdose prevention and response training programme for injection drug users in the Skid Row area of Los Angeles, CA. Int J Drug Policy 2010 5;21(3):186–93. 10.1016/j.drugpo.2009.01.003 19268564 PMC4291458

[13] Albert S, Brason FW, Sanford CK, Dasgupta N, Graham J, Lovette B. Project Lazarus: community-based overdose prevention in rural North Carolina. Pain Med 2011 6;12 Suppl 2:S77–S85. 10.1111/j.1526-4637.2011.01128.x 21668761

[14] Walley AY, Doe-Simkins M, Quinn E, Pierce C, Xuan Z, Ozonoff A. Opioid overdose prevention with intranasal naloxone among people who take methadone. J Subst Abuse Treat 2013 2;44(2):241–7. 10.1016/j.jsat.2012.07.004 22980450

[15] Green TC, Heimer R, Grau LE. Distinguishing signs of opioid overdose and indication for naloxone: an evaluation of six overdose training and naloxone distribution programs in the United States. Addiction 2008 6;103(6):979–89. 10.1111/j.1360-0443.2008.02182.x 18422830 PMC3163671

[16] Enteen L, Bauer J, McLean R, Wheeler E, Huriaux E, Kral AH, et al Overdose prevention and naloxone prescription for opioid users in San Francisco. J Urban Health 2010 12;87(6):931–41. 10.1007/s11524-010-9495-8 20967505 PMC3005091

[17] Community-based opioid overdose prevention programs providing naloxone—United States, 2010. MMWR Morb Mortal Wkly Rep 2012 2 17;61(6):101–5. 22337174 PMC4378715

[18] Walley AY, Xuan Z, Hackman HH, Quinn E, Doe-Simkins M, Sorensen-Alawad A, et al Opioid overdose rates and implementation of overdose education and nasal naloxone distribution in Massachusetts: interrupted time series analysis. BMJ 2013;346:f174 10.1136/bmj.f174 23372174 PMC4688551

[19] Davis CS, Ruiz S, Glynn P, Picariello G, Walley AY. Expanded access to naloxone among firefighters, police officers, and emergency medical technicians in Massachusetts. Am J Public Health 2014 8;104(8):e7–e9. 10.2105/AJPH.2014.302062 24922133 PMC4103249

[20] Ray B, O'Donnell D, Kahre K. Police officer attitudes towards intranasal naloxone training. Drug Alcohol Depend 2015 1 1;146:107–10. 10.1016/j.drugalcdep.2014.10.026 25468814

[21] Bazazi AR, Zaller ND, Fu JJ, Rich JD. Preventing opiate overdose deaths: examining objections to take-home naloxone. J Health Care Poor Underserved 2010 11;21(4):1108–13. 10.1353/hpu.2010.0935 21099064 PMC3008773

[22] Beletsky L, Rich JD, Walley AY. Prevention of fatal opioid overdose. JAMA 2012 11 14;308(18):1863–4. 10.1001/jama.2012.14205 23150005 PMC3551246

[23] Tobin KE, Davey MA, Latkin CA. Calling emergency medical services during drug overdose: an examination of individual, social and setting correlates. Addiction 2005 3;100(3):397–404. 15733253 10.1111/j.1360-0443.2005.00975.x

[24] Davidson PJ, McLean RL, Kral AH, Gleghorn AA, Edlin BR, Moss AR. Fatal heroin-related overdose in San Francisco, 1997–2000: a case for targeted intervention. J Urban Health 2003 Jun;80(2):261–73. 12791802 10.1093/jurban/jtg029 PMC3456286

[25] Burris S, Beletsky L, Castagna C, Casey C, Colin C, McLaughlin JM. Stopping an invisible epidemic: Legal issues in the provision of naloxone to prevent opioid overdose. Drexel Law Review 2009;1(2):273–339.

[26] Hewlett L, Wermeling DP. Survey of naloxone legal status in opioid overdose prevention and treatment. J Opioid Manag 2013 9;9(5):369–77. 10.5055/jom.2013.0179 24353049

[27] Beletsky L, Ruthazer R, Macalino GE, Rich JD, Tan L, Burris S. Physicians' knowledge of and willingness to prescribe naloxone to reverse accidental opiate overdose: challenges and opportunities. J Urban Health 2007 1;84(1):126–36. 17146712 10.1007/s11524-006-9120-z PMC2078257

[28] The Network for Public Health Law. Legal interventions to reduce overdose mortality: Naloxone access and overdose Good Samaritan laws. St. Paul, MN; 2014 Nov. http://www.networkforphl.org/_asset/qz5pvn/network-naloxone-10-4.pdf

[29] Davis CS, Southwell JK, Niehaus VR, Walley AY, Dailey MW. Emergency medical services naloxone access: a national systematic legal review. Acad Emerg Med 2014 10;21(10):1173–7. 10.1111/acem.12485 25308142

[30] Kimber J, Copeland L, Hickman M, Macleod J, McKenzie J, De AD, et al Survival and cessation in injecting drug users: prospective observational study of outcomes and effect of opiate substitution treatment. BMJ 2010;341:c3172 10.1136/bmj.c3172 20595255 PMC2895695

[31] Mattick RP, Breen C, Kimber J, Davoli M. Methadone maintenance therapy versus no opioid replacement therapy for opioid dependence. Cochrane Database Syst Rev 2009;(3):CD002209. 19588333 10.1002/14651858.CD002209.pub2 PMC7097731

[32] Mattick RP, Breen C, Kimber J, Davoli M. Buprenorphine maintenance versus placebo or methadone maintenance for opioid dependence. Cochrane Database Syst Rev 2014;2:CD002207. 10.1002/14651858.CD002207.pub4 PMC10617756 24500948

[33] MacArthur GJ, Minozzi S, Martin N, Vickerman P, Deren S, Bruneau J, et al Opiate substitution treatment and HIV transmission in people who inject drugs: systematic review and meta-analysis. BMJ 2012;345:e5945 10.1136/bmj.e5945 23038795 PMC3489107

[34] Barry CL, McGinty EE, Pescosolido BA, Goldman HH. Stigma, discrimination, treatment effectiveness, and policy: public views about drug addiction and mental illness. Psychiatr Serv 2014 10;65(10):1269–72. 10.1176/appi.ps.201400140 25270497 PMC4285770

[35] Burstein P. The impact of public opinion on public policy: A review and an agenda. Polit Res Quart 2003 3 1;56(1):29–40.

[36] Morone JA. Enemies of the people: the moral dimension to public health. J Health Polit Policy Law 1997 8;22(4):993–1020. 9334916 10.1215/03616878-22-4-993

[37] O'Keefe DJ. How to handle opposing arguments in persuasive messages: A meta-analytic review of the effects of one-sided and two-sided messages. Communications Yearbook 2015;22:209–49.

[38] Gross K. Framing Persuasive Appeals: Episodic and Thematic Framing, Emotional Response, and Policy Opinion. Political Psychology 2008 4 1;29(2):169–92.

[39] Kreuter MW, Green MC, Cappella JN, Slater MD, Wise ME, Storey D, et al Narrative communication in cancer prevention and control: a framework to guide research and application. Ann Behav Med 2007 6;33(3):221–35. 17600449 10.1007/BF02879904

[40] GfK. KnowledgePanel Design Summary. Palo Alto, CA; 2013. http://www.gfk.com/Documents/GfK-KnowledgePanel-Design-Summary.pdf

[41] Squiers LB, Bann CM, Dolina SE, Tzeng J, McCormack L, Kamerow D. Prostate-specific antigen testing: men's responses to 2012 recommendation against screening. Am J Prev Med 2013 8;45(2):182–9. 10.1016/j.amepre.2013.04.005 23867025

[42] Heckman CJ, Darlow S, Manne SL, Kashy DA, Munshi T. Correspondence and correlates of couples' skin cancer screening. JAMA Dermatol 2013 7;149(7):825–30. 10.1001/jamadermatol.2013.515 23864084 PMC3802540

[43] Barry CL, Brescoll VL, Brownell KD, Schlesinger M. Obesity metaphors: how beliefs about the causes of obesity affect support for public policy. Milbank Q 2009 3;87(1):7–47. 10.1111/j.1468-0009.2009.00546.x 19298414 PMC2879183

[44] Rothman EF, Edwards EM, Heeren T, Hingson RW. Adverse childhood experiences predict earlier age of drinking onset: results from a representative US sample of current or former drinkers. Pediatrics 2008 8;122(2):e298–e304. 10.1542/peds.2007-3412 18676515

[45] Davis MM, Fant K. Coverage of vaccines in private health plans: what does the public prefer? Health Aff (Millwood) 2005 5;24(3):770–9. 15886172 10.1377/hlthaff.24.3.770

[46] Mutz D. Population-Based Survey Experiments. Princeton: Princeton University Press; 2011.

[47] Des Jarlais DC, Paone D, Friedman SR, Peyser N, Newman RG. Regulating controversial programs for unpopular people: methadone maintenance and syringe exchange programs. Am J Public Health 1995 11;85(11):1577–84. 7485676 10.2105/ajph.85.11.1577 PMC1615684

[48] Burris S, Strathdee SA. To serve and protect? Toward a better relationship between drug control policy and public health. AIDS 2006 1 2;20(1):117–8. 16327327 10.1097/01.aids.0000194806.81917.19

[49] Paone D, Des Jarlais DC, Gangloff R, Milliken J, Friedman SR. Syringe exchange: HIV prevention, key findings, and future directions. Int J Addict 1995;30(12):1647–83. 8557411 10.3109/10826089509104419

