# Supplementary material for: Messaging to Increase Public Support for Naloxone Distribution Policies in the United States: Results from a Randomized Survey Experiment
Source: PLoS One. 2015 Jul 1;10(7):e0130050. doi: 10.1371/journal.pone.0130050 (PMC4488484; doi:10.1371/journal.pone.0130050)
Supplement: S1 Appendix — (DOCX) [file pone.0130050.s001.docx]

Appendix S1. Introductory text for the randomized survey experiment providing examples of opioid analgesics

Prescription pain medications are narcotic medications prescribed by a doctor to treat pain. These do not include "over-the-counter" pain relievers such as aspirin, Tylenol, or Advil that can be bought in drug stores or grocery stores without a doctor's prescription. To see examples of prescription pain medications, please click here.

[MOUSE OVER POPUP WINDOW FOR WORD “here”]

VICODIN®, LORTAB®, NORCO®, OR LORCET®/LORCET PLUS®, PERCOCET®, PERCODAN®, OR TYLOX®, OXYCONTIN®, HYDROCODONE, MORPHINE, KADIAN®, OXYCODONE, TRAMADOL, ULTRAM®, CODEINE, TYLENOL® WITH CODEINE, METHADONE, DILAUDID®, FIORICET®, FIORINAL®, OPANA®, OXYMORPHONE, BUPRENORPHINE, SUBOXONE, SUBUTEX, DARVOCET-N®, DARVON®, OR PROPOXYPHENE, DEMEROL®
