# Supplementary material for: Messaging to Increase Public Support for Naloxone Distribution Policies in the United States: Results from a Randomized Survey Experiment
Source: PLoS One. 2015 Jul 1;10(7):e0130050. doi: 10.1371/journal.pone.0130050 (PMC4488484; doi:10.1371/journal.pone.0130050)
Supplement: S2 Appendix — (DOCX) [file pone.0130050.s002.docx]

S2 Appendix. Development of a policy support and belief scale and effects of different message exposures on scaled outcomes

Cronbach’s alpha for policy questions: 0.7669

Cronbach’s alpha for belief questions: 0.6907

In factor analysis, all policy questions loaded on a single factor (Eigenvalue = 1.98642) and all belief questions also loaded on a single factor (Eigenvalue = 1.56643). The factor loadings for all questions in each scale were ≥ 0.45. No questions were deleted from the scales.

Next, we calculated the belief and policy support scales by averaging the responses to the questions, after reverse coding the negatively worded questions. We then created linear regression models with the policy support or belief scale as the dependent variable and message exposure as the independent variable of interest.

| Effect of messages on a policy support scale and a belief scale compared to the no-exposure control group | | | | | |
| --- | --- | --- | --- | --- | --- |
|  | Message Exposure | | | | |
| Scale^a^ | Factual information  (n = 260) | Factual information plus refutation  (n = 266) | Sympathetic narrative  (n = 264) | Sympathetic narrative plus factual information  (n = 276) | Sympathetic narrative plus factual information plus refutation  (n = 265) |
| Policy support  (n = 1580) | 0.27 (0.09 to 0.45)** | 0.53 (0.35 to 0.70)*** | 0.43 (0.27 to 0.59)*** | 0.76 (0.59 to 0.93)*** | 0.87 (0.69 to 1.05)*** |
| Belief  (n = 1587) | 0.39 (0.20 to 0.58)*** | 0.44 (0.25 to 0.63)*** | 0.48 (0.29 to 0.67)*** | 0.90 (0.72 to 1.08)*** | 0.85 (0.67 to 1.04)*** |
| *P ≤ 0.05 compared to the no-exposure control group using linear regression  ** P ≤ 0.01 compared to the no-exposure control group using linear regression  *** P ≤ 0.001 compared to the no-exposure control group using linear regression  ^a^Scales for policy support and beliefs consist of averaging responses to all policy and belief questions, respectively, after reverse coding negatively worded questions. | | | | | |
