# Supplementary figures and images for: Messaging to Increase Public Support for Naloxone Distribution Policies in the United States: Results from a Randomized Survey Experiment
Source: PLoS One. 2015 Jul 1;10(7):e0130050. doi: 10.1371/journal.pone.0130050 (PMC4488484; doi:10.1371/journal.pone.0130050)

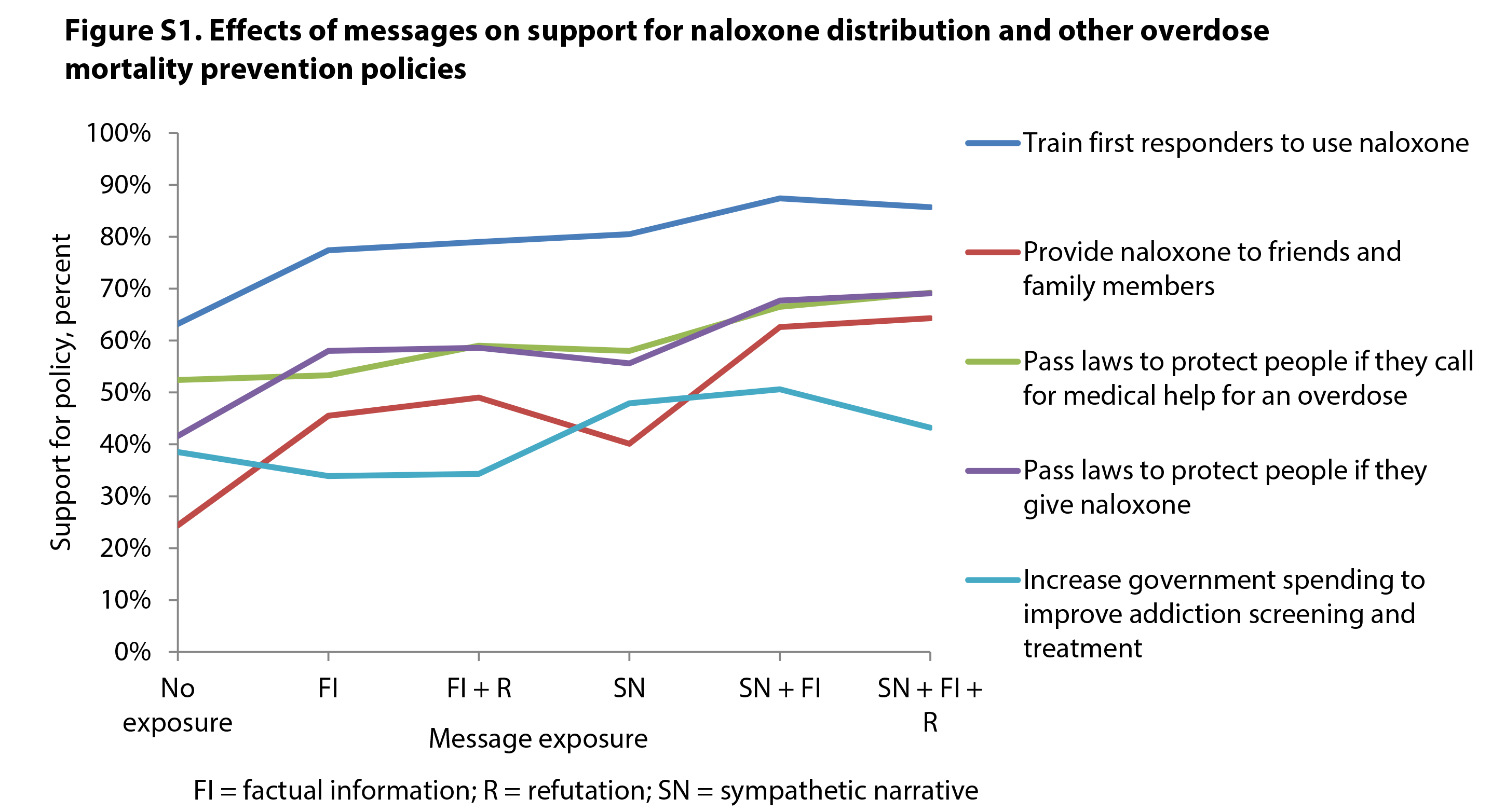

Supplement: S1 Fig — (TIF) [file pone.0130050.s003.tif]

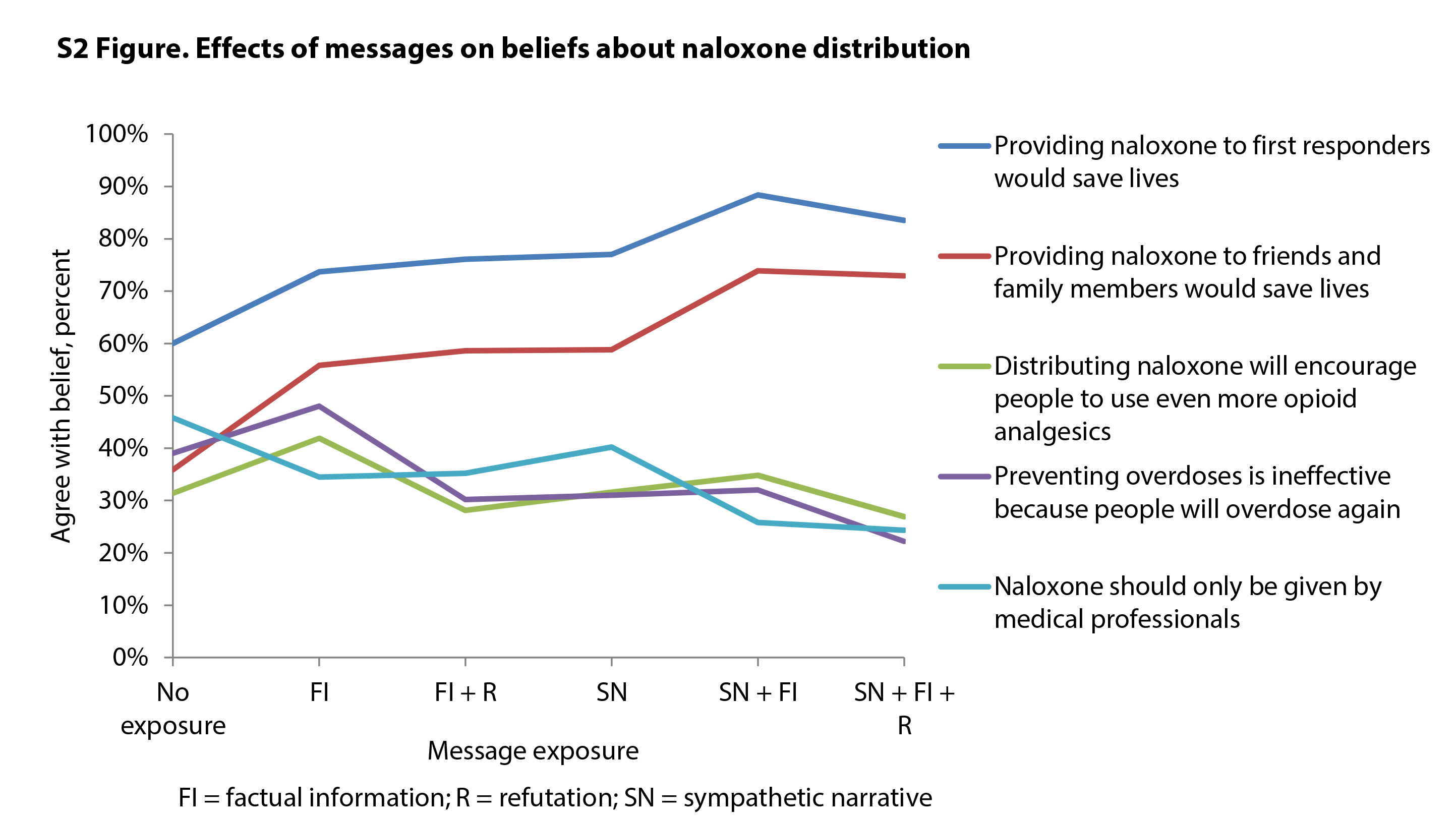

Supplement: S2 Fig — (TIF) [file pone.0130050.s004.tif]
